# *Apc* Mutation Enhances PyMT-Induced Mammary Tumorigenesis

**DOI:** 10.1371/journal.pone.0029339

**Published:** 2011-12-22

**Authors:** Jenifer R. Prosperi, Andrey I. Khramtsov, Galina F. Khramtsova, Kathleen H. Goss

**Affiliations:** 1 Department of Surgery, University of Chicago, Chicago, Illinois, United States of America; 2 Department of Medicine, University of Chicago, Chicago, Illinois, United States of America; Northwestern University Feinberg School of Medicine, United States of America

## Abstract

The *Adenomatous Polyposis Coli* (*APC*) tumor suppressor gene is silenced by hypermethylation or mutated in up to 70% of human breast cancers. In mouse models, *Apc* mutation disrupts normal mammary development and predisposes to mammary tumor formation; however, the cooperation between APC and other mutations in breast tumorigenesis has not been studied. To test the hypothesis that loss of one copy of APC promotes oncogene-mediated mammary tumorigenesis, *Apc^Min/+^* mice were crossed with the mouse mammary tumor virus (MMTV)-Polyoma virus middle T antigen (PyMT) or MMTV-c-Neu transgenic mice. In the PyMT tumor model, the *Apc^Min/+^* mutation significantly decreased survival and tumor latency, promoted a squamous adenocarcinoma phenotype, and enhanced tumor cell proliferation. In tumor-derived cell lines, the proliferative advantage was a result of increased FAK, Src and JNK signaling. These effects were specific to the PyMT model, as no changes were observed in MMTV-c-Neu mice carrying the *Apc^Min/+^* mutation. Our data indicate that heterozygosity of *Apc* enhances tumor development in an oncogene-specific manner, providing evidence that APC-dependent pathways may be valuable therapeutic targets in breast cancer. Moreover, these preclinical model systems offer a platform for dissection of the molecular mechanisms by which *APC* mutation enhances breast carcinogenesis, such as altered FAK/Src/JNK signaling.

## Introduction

Mutation of the *Adenomatous Polyposis Coli* (*APC*) tumor suppressor has been implicated in breast cancer; yet how it cooperates with oncogenic events to drive breast cancer pathogenesis is unknown. Numerous studies have demonstrated *APC* mutation or loss in human sporadic breast cancer (reviewed in [1]). APC inactivation has been reported in 5–70% of tumors, varying widely because of power constraints, the type of analysis performed, and specific molecular subtypes examined (reviewed in [1]). Unlike colorectal cancer where mutation is the primary mechanism for APC loss, APC expression is frequently down-regulated through transcriptional silencing via gene promoter methylation in breast cancer. For example, *APC* promoter hypermethylation has been observed in approximately 70% of inflammatory human breast tumors [2] and in 7% of metaplastic breast carcinomas [3]. *APC* promoter methylation also correlates with decreased disease-free and overall survival [4], providing evidence that APC down-regulation is not a passive bystander but, rather, contributes significantly to breast cancer.

Mouse models have further supported the importance of APC in breast cancer. Female mice carrying a germline heterozygous nonsense mutation in *Apc*, designated *Apc^Min/+^*, develop spontaneous and carcinogen- or radiation-induced mammary tumorigenesis, although spontaneous mammary tumors develop at a low frequency [5], [6], [7]]. Our laboratory demonstrated that mammary hyperplasia and squamous metaplasia are common in *Apc^Min/+^* mice, and APC is required for normal lobuloaloveolar development during pregnancy and lactation [8]. Other alleles, including *Apc^1638^*, also develop spontaneous mammary tumors and are sensitive to radiation or chemical carcinogens [9], [10]. Spontaneous tumors in *Apc^Δ474^* mice resemble squamous cell carcinomas [11], a pathological phenotype that has been observed often in mammary tumors in which Wnt/β-catenin signaling is aberrantly activated [12]. While whey acidic protein (WAP)- and β-lactoglobulin-cre-induced *Apc* deletion increased squamous metaplasias [13], [14], keratin-14 (K-14)-mediated *Apc* deletion led to mammary tumor formation [14], suggesting that the cell type in which APC is inactivated is critical for the associated phenotypes. Recently, truncation at codon 1572 in APC (*Apc^1572T^*) was shown to be sufficient for metastatic mammary tumors that resemble human metaplastic breast cancers [15].

It is now appreciated that most breast cancers contain more than 80 non-silent mutations [16]. These tumor models have addressed the importance of *Apc* mutation or deletion alone in mammary tumor initiation, but not the contribution of APC loss in the context of other oncogenic events. In this study, we addressed how *Apc* mutation cooperates with known breast cancer oncogenes to mimic more closely human sporadic breast cancer using mouse models. MMTV-PyMT transgenic females develop metastatic mammary tumors with high penetrance [17]. Despite the rapid development of aggressive tumors using this model, multiple studies have demonstrated the ability to enhance tumorigenesis [18], [19], [20]. Tumor progression in the MMTV-PyMT model follows a pattern similar to human sporadic breast cancer [21]. Further, signaling pathways activated downstream of PyMT overexpression, including the Src family of kinases, are commonly dysregulated in human breast cancers [22]. MMTV-c-Neu mammary tumors mimic HER2-positive human breast cancer and develop with lower penetrance and significantly longer latency [23]. The current study demonstrates that *Apc* mutation enhances PyMT-, but not Neu- induced mammary tumorigenesis, where it drives the squamous adenocarcinoma phenotype and provides a significant proliferation advantage. Importantly, these differences are associated with increased signaling through the FAK/Src/JNK network. These data support a model in which *APC* mutation may promote breast cancer by cooperating with other oncogenic changes to alter their histopathological and growth characteristics via the activation of signaling pathways downstream of cell-matrix interactions.

## Methods

### Ethics Statement

All procedures were performed with prior approval (protocol #71828) from the University of Chicago Institutional Animal Care and Use Committee, which has full accreditation from the Association for Assessment and Accreditation of Laboratory Animal Care.

### Mice

The MMTV-PyMT (line #634) and MMTV-c-Neu (line #202) transgenic mice were obtained from the Mouse Models of Human Cancers Consortium at the National Cancer Institute on a FVB/N background. *Apc^Min/+^* mice (The Jackson Laboratory, Bar Harbor, ME), maintained on a C57Bl/6 background, were backcrossed onto FVB/N to the N_6_ generation resulting in *Apc^Min/+^* mice expected to be >90% derived from FVB/N. Female *Apc^Min/+^* (FVB/N) mice were crossed to male MMTV-PyMT or MMTV-c-Neu mice to generate experimental (MMTV-PyMT;*Apc^Min/+^* or MMTV-c-Neu;*Apc^Min/+^*) and age-matched control (MMTV-PyMT;*Apc^+/+^* or MMTV-c-Neu;*Apc^+/+^*) N_6_F_1_ virgin females. Numbers of animals used for each experiment are specified in the figure legends.

### Analysis of mammary tumors and lung metastases

Mice were checked bi-weekly for palpable mammary tumors, and tumors were measured with calipers every other day. Tumor volume was calculated using the equation (mm^3^) = (a^2^×b)/2. For the survival study, animals were euthanized when tumors became ulcerated or tumor size reached 10% of body weight. Animals were injected with 75 mg/kg 5-bromo-2-deoxyuridine (BrdU, BD Biosciences, San Diego, CA) two hours prior to euthanasia at which time primary mammary tumors were harvested, cultured (see below) or fixed in 3.7% formaldehyde. Overt metastases were quantitated by visual inspection after the lungs were inflated with 3.7% formaldehyde. Surface lung metastases (>1 mm) were counted using a dissection microscope at 1.6× magnification. Paraffin-embedded sections were stained with hematoxylin and eosin (H&E) and evaluated by two individual blinded pathologists (AK and GK). Lungs were sectioned, stained with H&E, and examined for micrometastases (10–100 tumor cells) by a blinded observer (LQ) using 15 sections from each animal (n = 5 per genotype).

### LOH analysis of *Apc*

Genomic DNA was isolated from two 4 µm-paraffin-embedded sections of tumors and normal mammary gland from *Apc^Min/+^* mice as previously described [24]. A 156-bp fragment of the *Apc* gene was amplified using the PCR conditions as described by Luongo et al [25]. PCR products were then digested with HindIII to differentiate the 123-bp wildtype and 144-bp *Apc^Min^* alleles, and products were subjected to agarose gel electrophoresis (4% agarose) as described [25], [26]. Band intensity was quantified using NIH ImageJ software; data are presented as a ratio of WT∶Min alleles.

### Immunohistochemistry (IHC) and immunofluorescence (IF)

IHC for total β-catenin was performed and quantified as described previously [27]. For IF, tissues were blocked with 0.2% nonfat dried milk, 2% bovine serum albumin and 0.3% Triton X-100 in PBS for 30 min. For *in vitro* IF studies, cells were fixed with 3.7% formaldehyde and permeabilized with 0.3% Triton X-100 for 15 min prior to staining with the following primary antibodies diluted 1∶400 in blocking buffer: anti-BrdU rat monoclonal antibody (Abcam, cat# 6326, Cambridge, MA); anti-β-catenin rabbit polyclonal antibody (Lab Vision Products, cat# RB-9035); anti-E-cadherin mouse monoclonal antibody (BD Biosciences, cat# 610181); or anti-p-FAK mouse monoclonal antibody (Santa Cruz Biotechnology, cat# sc-81493, Santa Cruz, CA). Expression was detected using conjugated secondary antibodies (1∶200 each; Invitrogen, Carlsbad, CA). For visualization of F-actin, cells were co-stained with fluorescently conjugated Phalloidin (Invitrogen). Slides were mounted with Fluoromount G with Hoechst. Terminal deoxynucleotidyl transferase biotin-dUTP nick end labeling (TUNEL) assays were performed using the ApopTag Plus Peroxidase *in Situ* Apoptosis Detection Kit (Chemicon, Temecula, CA). For quantification, at least 200 cells were counted per tissue section, and sections from one tumor per mouse (n = 5 per genotype) were analyzed at each time point. The percentage of positive cells was averaged, and statistical analyses were performed using a Student's t-test.

### Cell culture and *in vitro* proliferation assay

Primary tumors were rinsed in sterile phosphate buffered saline (PBS), minced, and plated in RPMI media with 10% FBS and 1% penicillin/streptomycin. After two days, the media was changed and cells were routinely passaged using 0.25% trypsin/EDTA. For analysis, all cells were between 20–30 passages, and morphology was monitored by phase-contrast microscopy. SW480 cells (ATCC, Manassas, VA), used as a positive control for reporter assays, were grown in DMEM with 10% FBS and 1% penicillin/streptomycin. 1×10^5^ cells were plated in 12-well plates for 24 h prior to the addition of 10 µM BrdU and treated with either 50 µM PP2 (Sigma, St. Louis, MO), 20 or 30 µM SP600125 (Sigma), a combination of PP2 and SP600125, or DMSO as a control. After 12 h, cells were fixed in 3.7% formaldehyde, and BrdU IF was performed as described above.

### Western blot analysis

Total protein was isolated from tumor-derived cell lines using lysis buffer (50 mM Tris pH 7.5, 150 mM NaCl, 0.5% NP-40, 1.0 mM EDTA, 0.2 mM PMSF, and 1× protease inhibitor cocktail (Fisher, Pittsburgh, PA)). 20 µg of protein was separated by SDS-PAGE (10% gel), and transferred onto Immobilon-P membrane (Fisher). Membranes were blocked for 1 hr in 1× TBS with 0.1% Tween 20 and 5% nonfat dried milk, followed by overnight incubation with primary antibodies diluted 1∶1000 in TBST with 1% BSA (β-actin – Sigma, cat# A1978; Src (cat# 2108), p-Src (cat# 2101), JNK (cat# 9252), and p-JNK (cat# 4668)– Cell Signaling, Danvers, MA; FAK – BD Biosciences, cat# 610087; p-FAK – Santa Cruz Biotechnology, cat# sc-81493). Secondary antibody (anti-mouse or anti-rabbit IgG-HRP, 1∶2000) incubations were performed for 1 hr in 1× TBST with 1% BSA, and blots were developed using the Immun-Star HRP kit (BioRad). Representative blots are shown from 3 experiments.

### β-catenin/TCF reporter assays

Cells were plated in triplicate and transfected using Lipofectamine 2000 (Invitrogen) with either pTOPflash or pFOPflash as previously described [28], and co-transfected with pRL-TK (Renilla luciferase-thymidine kinase; Promega, Madison, WI). Lysates were harvested after 48 h and analyzed using the Dual Luciferase Assay System kit (Promega). Luciferase activity was normalized for transfection efficiency and FOPflash activity; the mean of 3 independent experiments is shown.

### Statistical analysis

Kaplan-Meier curves were generated for tumor latency and survival analysis, and statistical significance was determined using the Wilcoxon test. Differences in tumor type and grade, as well as β-catenin localization, were analyzed using the Fisher's exact test. For tumor size, an exact binomial goodness-of-fit test was performed. Student's t-test was used for statistical analysis of all remaining studies.

## Results

### *Apc^Min/+^* mutation decreases mammary tumor latency and survival in the PyMT model

**Figure 1:**
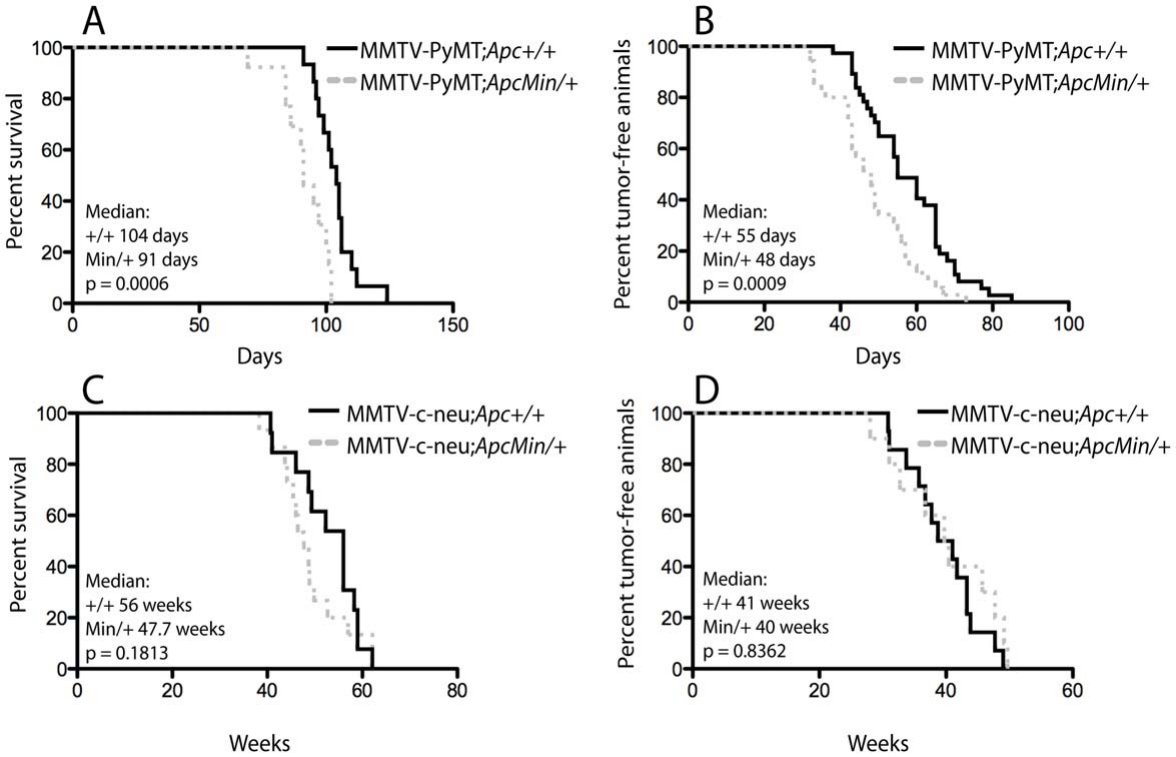
*Apc* heterozygosity accelerates MMTV-PyMT-mediated mammary tumorigenesis with no effect in the MMTV-c-Neu model. Experimental (MMTV-PyMT;*Apc^Min/+^* (n = 35 for survival and n = 13 for latency) and MMTV-c-Neu;*Apc^Min/+^* (n = 13)) and control (MMTV-PyMT;*Apc^+/+^* (n = 37 for survival and n = 15 for latency) and MMTV-c-Neu;*Apc^+/+^* (n = 15)) mice were palpated bi-weekly for tumor development. For the survival study, animals were sacrificed when the tumor reached 10% of the animal's body weight or became ulcerated. Kaplan-Meier curves for MMTV-PyMT tumor studies demonstrate that both (**A**) survival and (**B**) latency have a significant shift to the left in the presence of the *Apc^Min/+^* mutation. However, Kaplan-Meier curves for (**C**) survival and (**D**) latency show no change in the MMTV-c-Neu model with introduction of the *Apc^Min/+^* mutation. Median values are given, and significance was determined using the Wilcoxon test.

The *Apc^Min^* allele was backcrossed onto FVB/N to the N_6_ generation resulting in *Apc^Min/+^* mice expected to be >90% derived from FVB/N and found to live more than a year without severe intestinal complications. In the timeframe of the current studies, *Apc^Min/+^* (FVB/N) females do not develop spontaneous mammary tumors and were used for breeding with MMTV-PyMT or MMTV-c-Neu transgenic males. Despite the aggressive nature of MMTV-PyMT mammary tumorigenesis, heterozygosity of *Apc* resulted in a significant decrease in the median survival time of 91 days as compared to 104 days in the MMTV-PyMT;*Apc^+/+^* mice (**Figure 1A**; p = 0.0006). In addition, the MMTV-PyMT;*Apc^Min/+^* females developed tumors earlier than control animals such that the median latency was decreased from 55 to 48 days (**Figure 1B**; p = 0.0009). To determine whether this was a specific effect on PyMT-mediated mammary tumorigenesis, we assessed whether *Apc^Min^* heterozygosity would impact MMTV-c-Neu-driven tumor development using an identical approach. Interestingly, *Apc* mutation did not significantly impact MMTV-c-Neu tumor latency or survival (**Figure 1C and D**).

### The *Apc^Min/+^* mutation alters PyMT tumor histopathology

**Figure 2:**
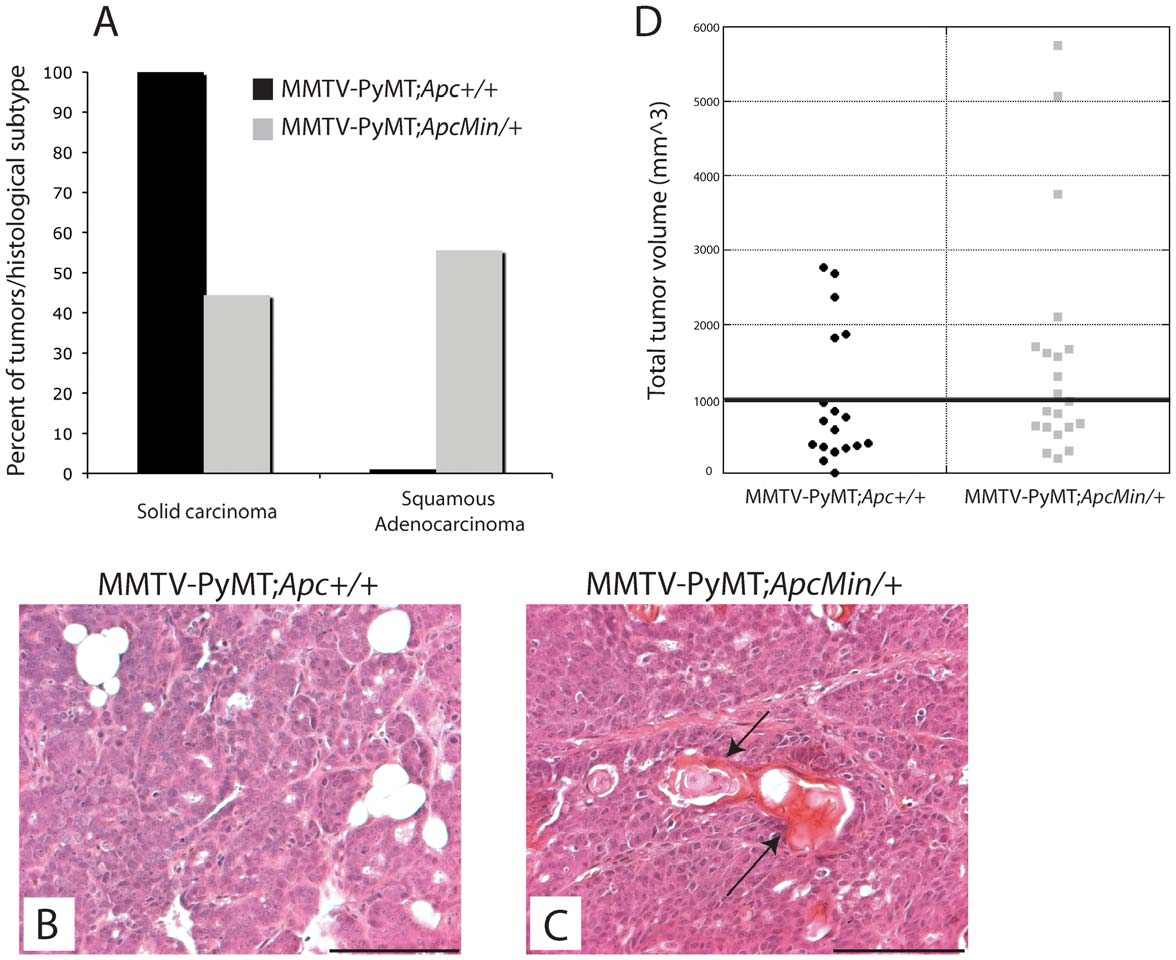
The *Apc^Min^* mutation alters the histopathology of MMTV-PyMT mammary tumors and enhances tumor volume. Tumors were fixed, embedded and stained with H&E. Quantification of the histopathological classification demonstrates that more tumors from MMTV-PyMT;*Apc^Min/+^* (n = 9) mice display a squamous adenocarcinoma phenotype rather than solid carcinomas in control animals (n = 7) (**A**, p<0.05 using Fishers exact test). Representative images of H&E-stained MMTV-PyMT;*Apc^+/+^* tumors showing development of solid carcinomas (**B**). In contrast, tumors from the MMTV-PyMT;*Apc^Min/+^* mice were predominately classified as squamous adenocarcinomas (**C**). Scale bar = 100 µm. (**D**) At 65 days of age, there was an increase in the number of MMTV-PyMT;*Apc^Min/+^* (n = 21) mice with a greater total tumor volume than the mean of the control animals (n = 18) (p<0.05 using exact binomial test goodness-of-fit).

Given that *Apc* mutation reduced overall animal survival in this initial study, tumorigenesis was then analyzed at 65 days of age to investigate early changes mediated by APC. All of the tumors (7/7) analyzed from the MMTV-PyMT;*Apc^+/+^* animals were identified as solid carcinomas (**Figure 2A**; H&E staining shown in **Figure 2B**); however, more than 50% of the MMTV-PyMT;*Apc^Min/+^* tumors (5/9) were classified as squamous adenocarcinomas (**Figure 2A**, p<0.05 with Fisher's exact test; H&E staining shown in **Figure 2C**). Despite this significant change, *Apc* heterozygosity did not affect the histopathology of MMTV-c-Neu mammary tumors; all tumors analyzed were solid carcinomas (5/5 mice per genotype; data not shown). Although there was not a significant increase in the mean tumor burden in the MMTV-PyMT;*Apc^Min/+^* mice, significantly more MMTV-PyMT;*Apc^Min/+^* mice (10/21) had tumor burden (i.e. total tumor volume per mouse) greater than the mean tumor burden (977.6±212 mm^3^) for the MMTV-PyMT;*Apc^+/+^* control mice at 65 days of age (**Figure 2D**; p≤0.05 by exact binomial goodness-of-fit test). No difference was observed in the average tumor number per mouse as MMTV-PyMT;*Apc^Min/+^* mice had 4.6 tumors/animal (n = 21) and MMTV-PyMT;*Apc^+/+^* mice had 3.8 tumors/animal (n = 18; p = 0.138). Moreover, neither overt (>1 mm) nor micro (10–100 cells) lung metastases were significantly affected by *Apc* mutation in either model (not shown).

A key step in intestinal tumorigenesis in *Apc^Min/+^* mice is loss of heterozygosity (LOH) of *Apc*, occurring in nearly 100% of tumors [25]. Therefore, we addressed whether mammary tumors isolated from MMTV-PyMT;*Apc^Min/+^* mice exhibited LOH of the wildtype *Apc* allele. Comparison of genomic DNA isolated from normal *Apc^Min/+^* mammary tissue using a PCR-based assay followed by quantification of the ratio of *Apc^+^* to *Apc^Min^* alleles demonstrated that mammary tumors from MMTV-PyMT;*Apc^Min/+^* mice generally retain the wildtype allele (**Figure S1**), suggesting that haploinsufficiency for *Apc* promotes tumor development in the MMTV-PyMT model.

### Proliferation and apoptosis are altered in MMTV-PyMT;*Apc^Min/+^* mammary tumors

**Figure 3:**
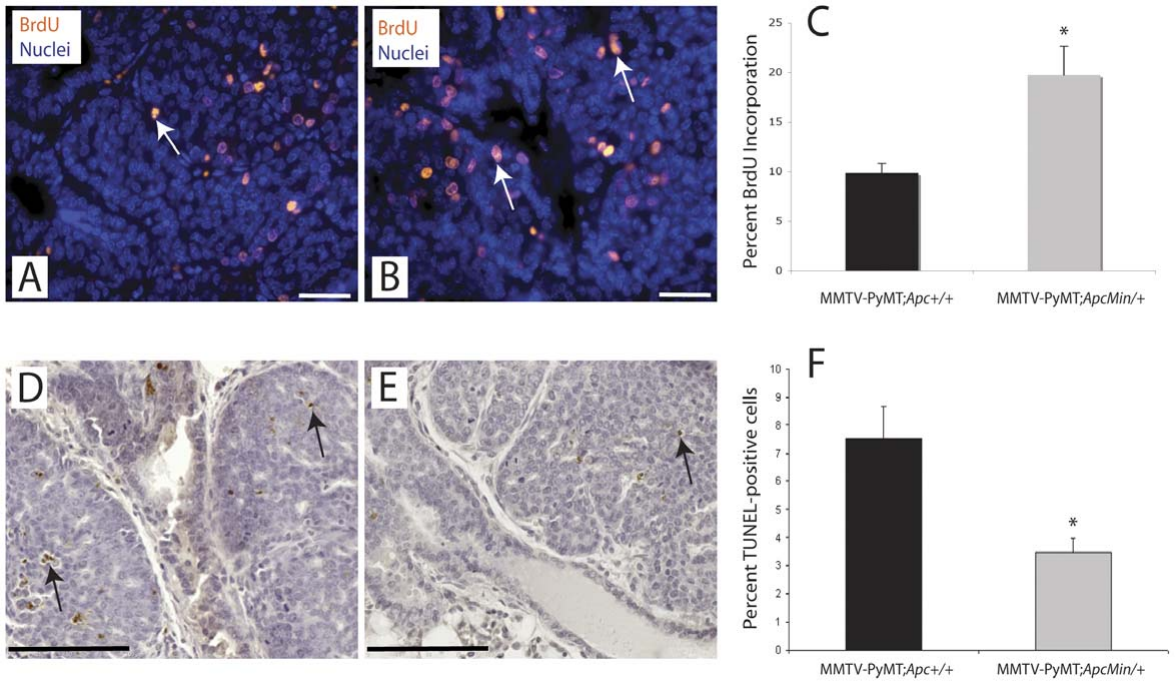
Introduction of *Apc^Min/+^* mutation promotes tumor cell proliferation and decreases apoptosis in MMTV-PyMT tumors. Animals were injected with BrdU 2 h prior to sacrifice and tumor sections were stained with an anti-BrdU antibody (red) to assess incorporation as a measure of S-phase entry. Nulcei were counterstained with Hoechst (blue). Apoptosis was quantified using TUNEL staining on tumor sections (brown); the tissues were counterstained with hematoxylin. Representative images of (**A and D**) MMTV-PyMT;*Apc^+/+^* (n = 12) and (**B and E**) MMTV-PyMT;*Apc^Min/+^* (n = 10) tumors are shown. Average percent BrdU incorporation (**C**) or percent TUNEL-positive cells (**F**) from both the survival and day 65 studies combined with standard error of the mean (SEM) shown. Scale bars = 20 µm for BrdU and 100 µm for TUNEL. *p<0.05 using a two-sided Student's t-test.

To identify the cellular basis for decreased tumor latency and survival in MMTV-PyMT;*Apc^Min/+^* mice, tumor cell apoptosis and proliferation were analyzed. Of note, tumors of both solid carcinoma and squamous adenocarcinoma phenotype were analyzed for subsequent studies. Proliferation in primary tumors from the survival study and at 65 days was measured by BrdU incorporation. We observed a statistically significant two-fold increase in the percentage of BrdU-positive cells in *Apc*-mutant mice (19.7% vs. 9.9% in control tumors; **Figure 3A–C**; p<0.05). In addition, TUNEL staining demonstrated a two-fold decrease in apoptotic cells in tumors from the MMTV-PyMT;*Apc^Min/+^* mice compared to control animals (3.5% compared to 7.5% in controls; **Figure 3D–F**; p<0.05). Collectively, these data indicate that APC inactivation enhances PyMT-mediated mammary tumorigenesis by simultaneously driving tumor cell proliferation and attenuating apoptosis.

### The Wnt/β-catenin pathway activation is not enhanced by *Apc* mutation in MMTV-PyMT mammary tumors

In colorectal cancer and other tumor types, APC inactivation is frequently associated with nuclear and cytosolic accumulation of β-catenin as a measure of canonical Wnt pathway activation [29]. To determine if APC mutation promoted mammary tumorigenesis via Wnt/β-catenin pathway stimulation, we next compared the subcellular localization of β-catenin in mammary tumors isolated from MMTV-PyMT;*Apc^+/+^* and MMTV-PyMT;*Apc^Min/+^* mice (**Figure S2**). In mammary tumors isolated from both genotypes, the majority of tumors maintained some β-catenin localization at the cell membrane. 2/4 (50%) of tumors from the MMTV-PyMT;*Apc^+/+^* mice demonstrated high levels (i.e. IHC score of 2 or 3) of either cytosolic or nuclear β-catenin, suggesting that these tumors show some basal Wnt/β-catenin pathway activity (**Figure S2A**). All MMTV-PyMT;*Apc^Min/+^* tumors (5/5) had high levels of cytosolic or nuclear β-catenin (**Figure S2A**) but a difference between genotypes was not statistically significant (p = 0.17 using Fisher's exact test). Analysis of the mRNA and protein expression of β-catenin target genes, *cyclin D1, axin2, and c-myc*, in tumors also did not show any difference between the two genotypes, although the expression of these genes were already significantly elevated in all of the tumors compared to normal mammary gland (not shown). These data suggest that *Apc* mutation does not induce Wnt/β-catenin pathway signaling over basal levels in MMTV-PyMT tumors.

### MMTV-PyMT;*Apc^Min/+^* tumor cells have altered morphology, proliferation, and signaling through the FAK/Src/JNK pathway

**Figure 4:**
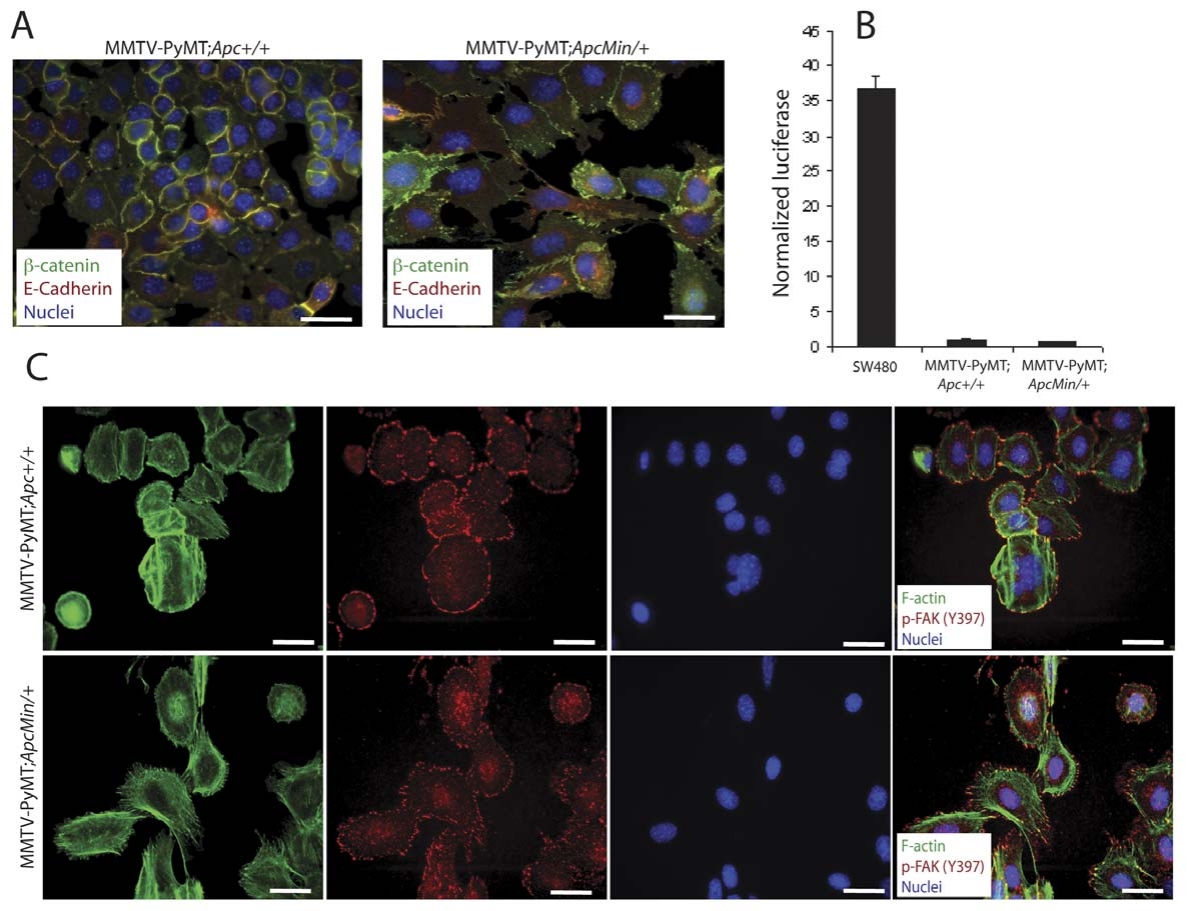
Cell lines generated from tumors have similar phenotypes to primary tumors. Primary cells were isolated from mammary tumors from MMTV-PyMT;*Apc^+/+^* and MMTV-PyMT;*Apc^Min/+^* mice. (**A**) IF with a β-catenin antibody (green) and E-cadherin (red) antibody shows restricted localization of both proteins to cell-cell contacts in the control cells (left panel). In the *Apc*-mutant cells (right panel), junctional β-catenin and E-cadherin are observed but β-catenin is also localized in a punctate pattern in the cytosol or membrane. No nuclear accumulation of β-catenin is observed. Nuclei are stained with Hoechst (blue). Scale bars = 20 µm. (**B**) β-catenin/TCF reporter assays showed minimal basal Wnt/β-catenin pathway activation in both cell lines. SW480 cells were used as a positive control. The data are shown as a ratio of normalized TOPflash∶FOPflash values. (**C**) IF staining with phalloidin to label F-actin (green) illustrates a more spread morphology with extensive lamellipodia formation for the MMTV-PyMT;*Apc^Min/+^* tumor cells (bottom panels) compared to the control cells (top panels). In addition, phospho-FAK (Tyr397, red) localizes in plaques primarily at the lamellipodia along actin stress fibers. Nuclei are stained with Hoechst (blue). Scale bars = 20 µm.

To identify the molecular mechanisms downstream of APC mutation responsible for promoting mammary tumorigenesis in the PyMT model, tumor cells were cultured from animals from each genotype. Two lines from each genotype were thoroughly characterized, although only one of each genotype is shown for space considerations (data from the second set is shown in **Figure S3**). IF for β-catenin demonstrated exclusive localization at cell-cell junctions in the MMTV-PyMT;*Apc^+/+^* tumor cells (**Figure 4A**). In the MMTV-PyMT;*Apc^Min/+^* cells, β-catenin was localized to cell-cell contacts as well as in punctate clusters in the cytosol or membrane; however, nuclear β-catenin localization was not observed (**Figure 4A**). This pattern of β-catenin was also coincident with a pronounced spread morphology in the MMTV-PyMT;*Apc^Min/+^* cells with fewer cell-cell interactions and more extensive lamellipodia (**Figure 4A**). This is in contrast to the more epithelial, cobblestone morphological appearance of the MMTV-PyMT;*Apc^+/+^* cells (**Figure 4A**). To assess active Wnt/β-catenin signaling, β-catenin/TCF reporter assays were performed in which cells were transiently transfected with a plasmid expressing multiple TCF-binding sites upstream of firefly luciferase (TOPflash) and normalized using a co-transfected renilla luciferase plasmid. Transfection with a plasmid containing mutant TCF sites (FOPflash) controlled for specificity. Both sets of tumor cells had very low basal reporter activity compared to the SW480 colorectal carcinoma cell line that carries an *APC* mutation, and no significant difference was observed between the cell lines (**Figure 4B**). Both types of tumor cells, however, were able to respond to pathway induction using exogenous Wnt ligand (not shown), suggesting that their low basal activity was not due to some selective inability to induce signaling.

**Figure 5:**
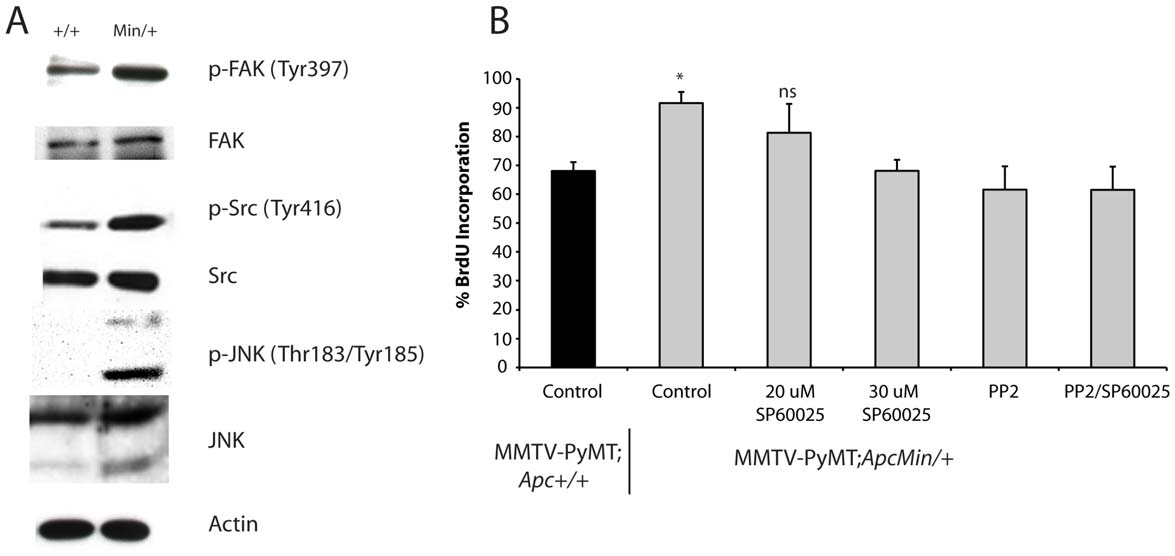
Increased cell proliferation in *Apc*-mutant tumor cells is dependent on active Src and JNK signaling. (**A**) Western blotting was performed on total cell lysates from the tumor cell lines with antibodies to detect total and phosphorylated forms of JNK, FAK and Src. Increased phosphorylation of JNK, FAK, and Src is observed in the MMTV-PyMT;*Apc^Min/+^* tumor cells compared to control cells. Actin was used as a loading control. (**B**) Tumor cell proliferation was assessed using BrdU incorporation and staining with an anti-BrdU antibody. Quantification shows that, consistent with the *in vivo* studies, MMTV-PyMT;*Apc^Min/+^* tumor cells have enhanced proliferation compared to control cells. Treatment of the MMTV-PyMT;*Apc^Min/+^* cells with an inhibitor of Src, a high dose of the JNK inhibitor (30 µM) or a combination of Src and JNK inhibitors for 12 h significantly inhibited their BrdU incorporation, reducing it to levels similar to control cells (*p<0.05 MMTV-PyMT;*Apc^Min/+^* cells compared to control, PP2-treated, and PP2/SP600125-treated cells).

To further investigate the morphological changes observed in the MMTV-PyMT;*Apc^Min+^* cells, focal adhesion morphology and distribution were analyzed. Localization of active, autophosphorylated FAK (Tyr 397), a critical signaling component of focal adhesions, was predominantly confined to the rounded cell perimeter of MMTV-PyMT;*Apc^+/+^* tumor cells (**Figure 4C**). In contrast, many of the MMTV-PyMT;*Apc^Min/+^* cells had phospho-FAK localized at the ends of the actin stress fibers in an elongated, plaque-like distribution pattern. The expanded lamellipodia and branched actin networks are especially apparent in the IF images of the phalloidin-stained MMTV-PyMT;*Apc^Min/+^* cells (**Figure 4C**). The enhanced spreading and pronounced focal adhesions in the cells carrying an *Apc* mutation was also consistent with a significant increase in phospho-FAK (Tyr397) in lysates from the MMTV-PyMT;*Apc^Min/+^* tumor cells by western blotting (**Figure 5A**). In addition, tumors from MMTV-PyMT;*Apc^Min/+^* mice showed more robust phospho-FAK (Tyr397) staining by IF than control tumors (**Figure S3E**).

One well-characterized consequence of PyMT overexpression in mammary tumorigenesis is activation of the Src family of kinases (reviewed in [22]). Additionally, Src is activated by FAK at focal adhesions (reviewed in [30]). As expected, Src activity, measured by phosphorylation at Tyr416, was detectable in MMTV-PyMT;*Apc^+/+^* tumor cells; yet, in the presence of the *Apc^Min/+^* mutation, phosphorylated Src was significantly up-regulated (**Figure 5A**). Because interactions between FAK and Src can activate the mitogen-activated protein kinase (MAPK), c-Jun N-terminal kinase (JNK) [30], JNK phosphorylation was examined. Like phospho-FAK and phospho-Src, an increase in phosphorylated (Thr183/Tyr185) JNK was observed in the MMTV-PyMT;*Apc^Min/+^* cells compared to control cells, which showed very little JNK activity (**Figure 5A**). Although total Src and JNK are increased slightly in the MMTV-PyMT;*Apc^Min/+^* tumor cells, this does not solely account for the increase in phosphorylated protein.

The FAK/Src/JNK signaling pathway is implicated in regulating proliferation downstream of cell-matrix interactions [31], [32]. Therefore, we speculated that increased Src or JNK activation might be responsible for the elevated proliferation in tumors from MMTV-PyMT;*Apc^Min/+^* mice. To confirm that the tumor cells maintained the proliferative advantage of the primary tumors, MMTV-PyMT;*Apc^Min/+^* cells were analyzed and showed an increased level of BrdU incorporation (91% vs 68% in the control cells; *p<0.05; **Figure 5B**). Next, the experimental and control cells were treated either with a JNK inhibitor, SP600125, a Src inhibitor, PP2, or a combination of both. The Src inhibitor or the combination resulted in a significant decrease of proliferation (62% BrdU incorporation) compared to vehicle-treated MMTV-PyMT;*Apc^Min/+^* cells (91%; *p<0.05; **Figure 5B**). In fact, BrdU incorporation in the MMTV-PyMT;*Apc^Min/+^* cells after PP2 or PP2/SP600125 treatment was statistically equivalent to the MMTV-PyMT;*Apc^+/+^* control cells without any treatment (68%; **Figure 5B**), suggesting that hyperactivation of Src and JNK signaling are partially responsible for the increased proliferation observed in the MMTV-PyMT;*Apc^Min/+^* cells. Consistent with the known basal activation of Src in MMTV-PyMT tumors ([33] and reviewed in [22]), proliferation was also decreased in control cells using both PP2 and the combination of PP2/SP600125 (data not shown). At the dose used here, the JNK inhibitor had no effect on proliferation of the MMTV-PyMT;*Apc^Min/+^*; however, the JNK inhibitor could significantly suppress proliferation at higher doses (**Figure 5B**). These results, taken together, suggest that one mechanism by which APC mutation promotes mammary tumor cell proliferation in the PyMT model is through activation of Src and JNK signaling downstream of FAK and enhanced tumor cell-matrix interactions.

## Discussion

We have demonstrated that *Apc* mutation cooperates with the PyMT oncogene to significantly accelerate mammary tumorigenesis and alter the tumor histopathology. It was unexpected that the effect of APC mutation would be specific to the MMTV-PyMT mammary tumor model. In fact, we had predicted that the MMTV-c-Neu model, which has a longer tumor latency and lifespan than MMTV-PyMT mice, might be more sensitive to the *Apc^Min/+^* mutation. Few studies have shown disparate effects in tumor development in the MMTV-c-Neu and MMTV-PyMT transgenic models. For example, MUC1 deficiency significantly attenuated PyMT tumorigenesis but had no effect on Neu-induced tumors [34], [35]. Because MUC1 localization is disrupted in mammary glands from *Apc^Min/+^* females [8], we investigated MUC1 expression in the PyMT animals but detected no difference (J.R.P., K.H.G., unpublished results). While a number of similarities between MMTV-PyMT and MMTV-c-Neu tumor models exist, there are also several important differences (reviewed in [36]). A wide array of pathways are activated downstream of PyMT, including PI3K and Shc, but most involve direct activation of Src kinases [22]. Activation of c-Src is required for PyMT-mediated tumorigenesis [33] but does not appear to be critical downstream of Neu [37]. Neu overexpression activates many signaling networks, including the PI3K and Wnt/β-catenin pathways (reviewed in [38], [39]). One possible explanation for oncogene-specific effects of *Apc*-mutation on tumorigenesis is that redundant signaling is activated by Neu expression and *Apc* mutation. We observed that Src was hyperactivated the MMTV-PyMT;*Apc^Min/+^* tumor cells, and this up-regulation was not due to elevated levels of PyMT itself in the *Apc^Min/+^* tumors (J.R.P., K.H.G., unpublished results). Modifiers that impact mammary tumor development in the *Apc^Min/+^* mice and influence PyMT-mediated tumorigenesis have been identified and mapped [40]. However, genetic background and the presence of such modifiers are not likely to be confounding factors in our studies since 1) both transgenic alleles were on FVB/N background, 2) the same backcrossed *Apc^Min/+^*(FVB) animals were used for breeding to both models, and 3) genetically matched controls were used for all analyses. Another unexpected finding was that *Apc* mutation did not impact lung metastases in either tumor model. Recently, *APC* deletions and nuclear β-catenin accumulation were linked to brain metastases [41], and Wnt signaling was implicated in breast cancer metastasis to the lung, brain and bone [42], [43], [44]. Mammary tumors in the *Apc1572T* genetic model are highly metastatic to the lung [15]. It is possible that the short tumor latency and survival in the PyMT model precluded us from observing a significant effect on lung metastasis or that the cooperative signaling pathways downstream of *Apc* mutation and PyMT or Neu involved in primary tumor development are distinct from metastasis mediators. Further detailed investigation into the role of APC in metastasis is necessary to discriminate between these possibilities.

In addition to accelerating tumorigenesis in the PyMT model, *Apc* mutation altered the tumor phenotype from solid carcinomas to squamous adenocarcinomas. Interestingly, multiple models of ectopic Wnt/β-catenin signaling in the mammary gland develop squamous mammary tumors or areas of transdifferentiation [8], [11], [13], [14], [15], [45], [46], [47], [48]. However, the histopathological changes we observed cannot be attributed solely to Wnt/β-catenin pathway activation since the basal activity in PyMT tumors was not significantly changed in *Apc*-mutant cells. Although a modest induction of Wnt/β-catenin activity is possible, we previously published the importance of Wnt-independent APC activities, such as regulation of apical-basal polarity and morphogenesis, in regulating lobuloalveolar development and the occurrence of squamous metaplasia in mammary glands from *Apc^Min/+^* mice [8]. Multiple signaling molecules are known to be involved in squamous differentiation of the breast, including SMAD4 [49], cyclin D1 [50], cyclin D3 [51], and JNK [52]. In the PyMT;*Apc^Min/+^* model, we observed increased phosphorylation of JNK (**Figure 5A**) without a change in cyclin D1 expression (J.R.P., K.H.G., unpublished results). Although JNK was implicated in squamous differentiation downstream of Wnt/β-catenin activation [52], APC loss, independent of Wnt/β-catenin signaling, could activate this pathway. Axin, another component of the β-catenin destruction complex, induces JNK through a Wnt-independent mechanism [53]. Some differences in tumor histopathology *in vivo* were retained in the *in vitro* cell cultures. Specifically, the *Apc*-mutant tumor cells had a more spread morphology, enhanced cell-matrix interactions with fewer cell-cell contacts. Hyperactivation of JNK has been demonstrated to be responsible for a partial epithelial-mesenchymal transition (EMT) and promoting cell survival [54]. These morphological changes are also consistent with alterations directly in FAK-mediated signaling. Subconfluent FAK^−/−^ fibroblasts have a rounded morphology and similar staining pattern of focal adhesion markers to the MMTV-PyMT;*Apc^+/+^* cells [55]. Collectively, these data suggest that the augmented JNK and FAK signaling observed in the MMTV-PyMT;*Apc^Min/+^* cells may be responsible for the morphological changes observed *in vivo* and *in vitro*.

The mechanisms responsible for increased tumor cell proliferation and decreased survival by APC inactivation are likely to be more difficult to fully dissect. We found elevated FAK phosphorylation, consistent with morphological changes observed and recent data showing that conditional knockout of *Apc* in the intestine results in a c-myc-dependent FAK up-regulation, which is necessary for the increased proliferation and transformation of the intestinal epithelium [56]. Since an increase in c-myc, or other Wnt/β-catenin target genes, was not observed in *Apc*-mutant tumors, it is important to consider alternative mechanisms by which APC mutation mediates FAK activation. APC at the leading edge of cells is required for directed cell motility [57], [58] and has been hypothesized to interact with integrins through its association with both the actin and microtubule cytoskeleton [59]. Disruption of these interactions, particularly leading to changes in actin or microtubule polymerization or dynamics, could perturb FAK signaling, and current studies in the laboratory are aimed at investigating this possibility. We also identified increased phosphorylation of Src (Tyr 416) and JNK (Thr183/Tyr185). Interestingly, FAK autophosphorylation recruits Src to the focal adhesions, where, through Rac activation, JNK and other signaling molecules, are activated [60]. In human breast cancer, FAK expression is correlated with phospho-Src (Tyr215) [61] and poor prognosis [62]. Therefore, we hypothesize that APC mutation increases FAK autophosphorylation to induce downstream signaling through Src and JNK. Like Src, previous studies have demonstrated that mammary-specific FAK deletion protects against MMTV-PyMT-driven tumors through inhibiting proliferation, and that autophosphorylation at Tyr397 is required for tumor development [63]. Furthermore, loss of either Src or FAK in mammary epithelial cells results in decreased proliferation in PyMT-induced mammary hyperplasias and tumors [64], [65]. Our findings using inhibitors of Src and JNK suggest that hyperactivation of these signaling pathways is involved in the enhanced proliferation resulting from *Apc* mutation. Although these studies have focused on *in vivo* models, the results are likely to be relevant to human breast cancer. The tumors that arise in these mice resemble metaplastic carcinomas of the breast in humans, cancers previously associated with APC down-regulation [3]. Our data also suggest that the targeted inhibition of the signaling pathways specifically hyper-activated downstream of APC mutation, such as Src and JNK, may prove to provide a therapeutic benefit for these cancers.

## Supporting Information

Figure S1 **LOH is not observed in mammary tumors from MMTV-PyMT;** ***Apc^Min/+^*** **mice.** Quantification of the wildtype and *Apc^Min^* alleles was determined in genomic DNA isolated from tumors (n = 6) and cell lines derived from the tumors using a PCR-based assay as described [27]. The ratio of the *Apc^+^* to the *Apc^Min^* bands was then quantified by densitometry. Controls included DNA isolated from tails, normal intestine, a colon tumor and normal (non-tumor) mammary gland from *Apc^Min/+^* mice. To verify that intensity of the bands for each allele correlated to the ratio, *Apc^+/+^* tail DNA was spiked with increasing concentrations of *Apc^Min/+^* genomic DNA (at a ratio of 1∶1, 1∶3, and 1∶7). As expected, while the intensity of the *Apc^Min^* band increased in a dose-dependent manner, the *Apc^+^* band and the corresponding ratio decreased. Mammary tumors from MMTV-PyMT;*Apc^Min/+^* mice, and cell lines derived from them, retain the *Apc^+^* allele.
(PDF)

Figure S2 **Alterations in MMTV-PyMT-driven tumorigenesis in the presence of the** ***Apc^Min^*** **mutation are not associated with Wnt/β-catenin pathway hyperactivation.** β-catenin localization and intensity were analyzed in MMTV-PyMT;*Apc^+/+^* (n = 4) and MMTV-PyMT;*Apc^Min/+^* (n = 5) tumor sections by IHC using an anti-β-catenin antibody as previously described [25]. Data are shown as the percent of tumors with positive staining for membrane, cytosolic, and nuclear β-catenin with staining intensity on the x-axis. (**A**) No significant difference is observed in membrane, cytosolic, or nuclear expression of β-catenin in tumors isolated from the MMTV-PyMT;*Apc^Min/+^* animals compared to controls. Representative images of β-catenin localization at the membrane (**B**, white arrows), cytosol (**C**, black arrows), or nucleus (**D**, black arrows) are shown. The magnified region in the inset is marked in each image by a hatched box. Scale bar = 50 µm.
(PDF)

Figure S3 **A second set of cell lines isolated from MMTV-PyMT;** ***Apc^+/+^*** **and MMTV-PyMT;** ***Apc^Min/+^*** **mice show similar phenotypes.** (**A**) IF with a β-catenin antibody (green) and E-cadherin (red) antibody shows restricted localization of both proteins to cell-cell contacts in the control cells (left panel). In the *Apc*-mutant cells (right panel), junctional β-catenin and E-cadherin are observed but β-catenin is also localized in a punctate pattern in the cytosol or membrane. No nuclear accumulation of β-catenin is observed. Nuclei are stained with Hoechst (blue). Scale bars = 20 µm. (**B**) β-catenin/TCF reporter assays showed little basal activity in both cell lines. SW480 cells were used as a positive control. The data are shown as a ratio of normalized TOPflash∶FOPflash values. (**C**) Western blotting was performed on total cell lysates from the tumor cell lines with antibodies to detect total and phosphorylated forms of JNK, FAK and Src. Increased phosphorylation of JNK, FAK, and Src is observed in the MMTV-PyMT;*Apc^Min/+^* tumor cells compared to control cells. (**D**) Tumor cell proliferation was assessed using BrdU incorporation and staining with an anti-BrdU antibody. Quantification shows that, consistent with the *in vivo* studies, MMTV-PyMT;*Apc^Min/+^* tumor cells have enhanced proliferation compared to control cells. (**E**) IF with a phospho-FAK (Tyr 397) antibody (green) shows increased expression in the tumors from MMTV-PyMT;*Apc^Min/+^* mice (right panel) compared to the control tumors (left panel). Nuclei are stained with Hoechst (blue). Scale bars = 20 µm.
(PDF)
