# Supplementary figures and images for: Apc Mutation Enhances PyMT-Induced Mammary Tumorigenesis
Source: PLoS One. 2011 Dec 22;6(12):e29339. doi: 10.1371/journal.pone.0029339 (PMC3245255; doi:10.1371/journal.pone.0029339)

Figure S1

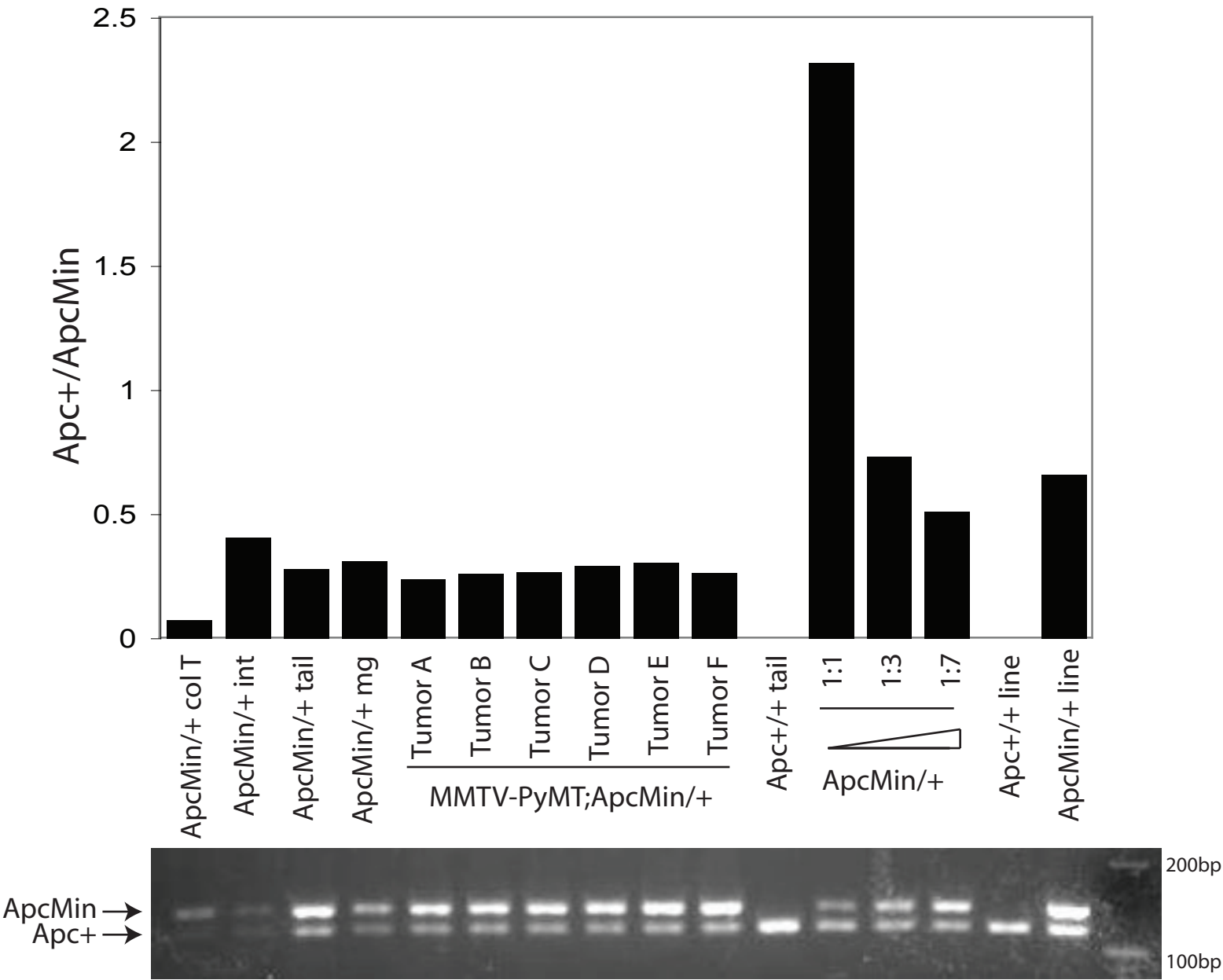

Supplement: Figure S1 — LOH is not observed in mammary tumors from MMTV-PyMT; ApcMin/+ mice. Quantification of the wildtype and ApcMin alleles was determined in genomic DNA isolated from tumors (n = 6) and cell lines derived from the tumors using a PCR-based assay as described [27]. The ratio of the Apc+ to the ApcMin bands was then quantified by densitometry. Controls included DNA isolated from tails, normal intestine, a colon tumor and normal (non-tumor) mammary gland from ApcMin/+ mice. To verify that intensity of the bands for each allele correlated to the ratio, Apc+/+ tail DNA was spiked with increasing concentrations of ApcMin/+ genomic DNA (at a ratio of 1∶1, 1∶3, and 1∶7). As expected, while the intensity of the ApcMin band increased in a dose-dependent manner, the Apc+ band and the corresponding ratio decreased. Mammary tumors from MMTV-PyMT;ApcMin/+ mice, and cell lines derived from them, retain the Apc+ allele. (PDF) [file pone.0029339.s001.pdf]

Figure S2

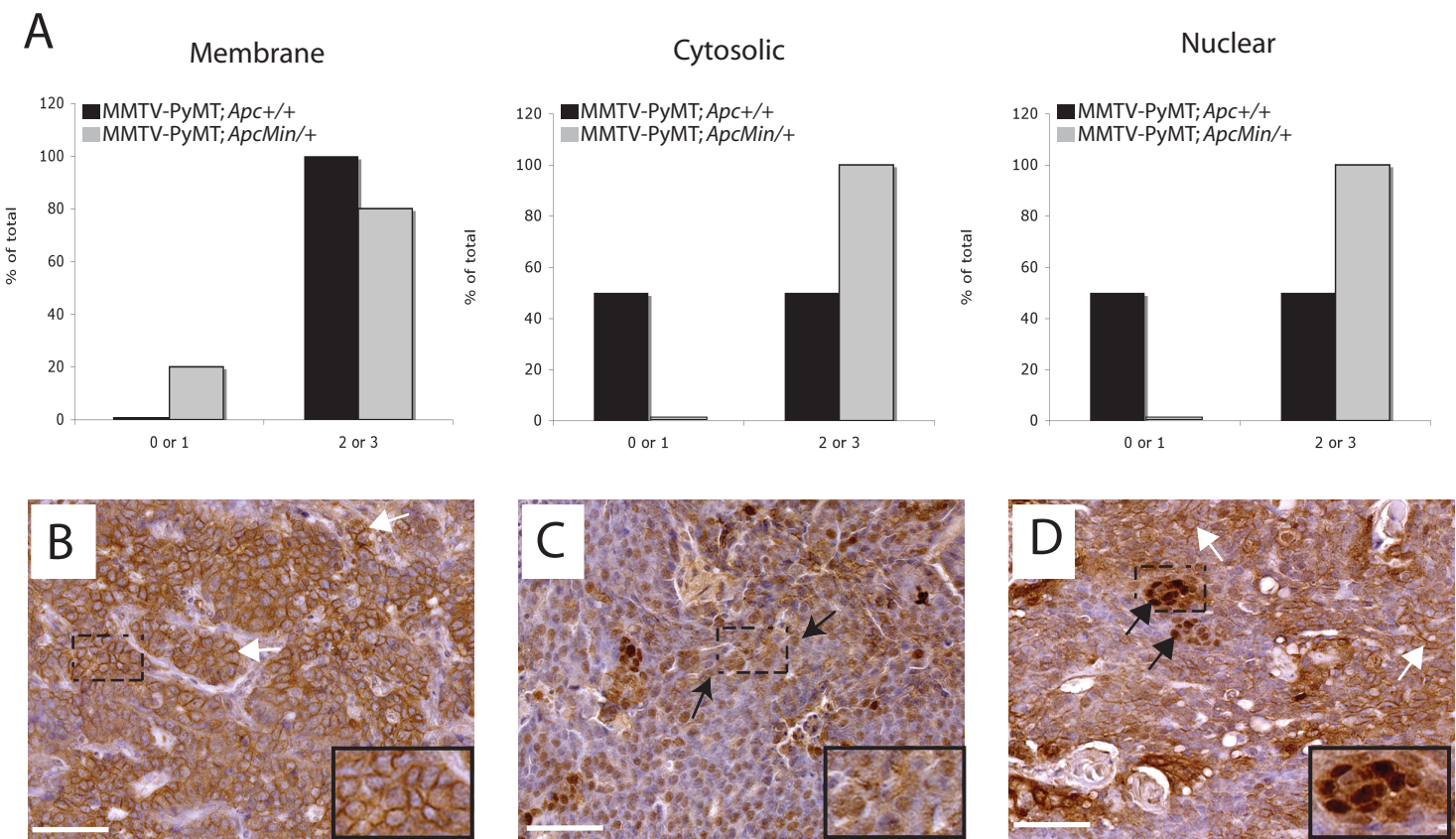

Supplement: Figure S2 — Alterations in MMTV-PyMT-driven tumorigenesis in the presence of the ApcMin mutation are not associated with Wnt/β-catenin pathway hyperactivation. β-catenin localization and intensity were analyzed in MMTV-PyMT;Apc+/+ (n = 4) and MMTV-PyMT;ApcMin/+ (n = 5) tumor sections by IHC using an anti-β-catenin antibody as previously described [25]. Data are shown as the percent of tumors with positive staining for membrane, cytosolic, and nuclear β-catenin with staining intensity on the x-axis. (A) No significant difference is observed in membrane, cytosolic, or nuclear expression of β-catenin in tumors isolated from the MMTV-PyMT;ApcMin/+ animals compared to controls. Representative images of β-catenin localization at the membrane (B, white arrows), cytosol (C, black arrows), or nucleus (D, black arrows) are shown. The magnified region in the inset is marked in each image by a hatched box. Scale bar = 50 µm. (PDF) [file pone.0029339.s002.pdf]

Figure S3

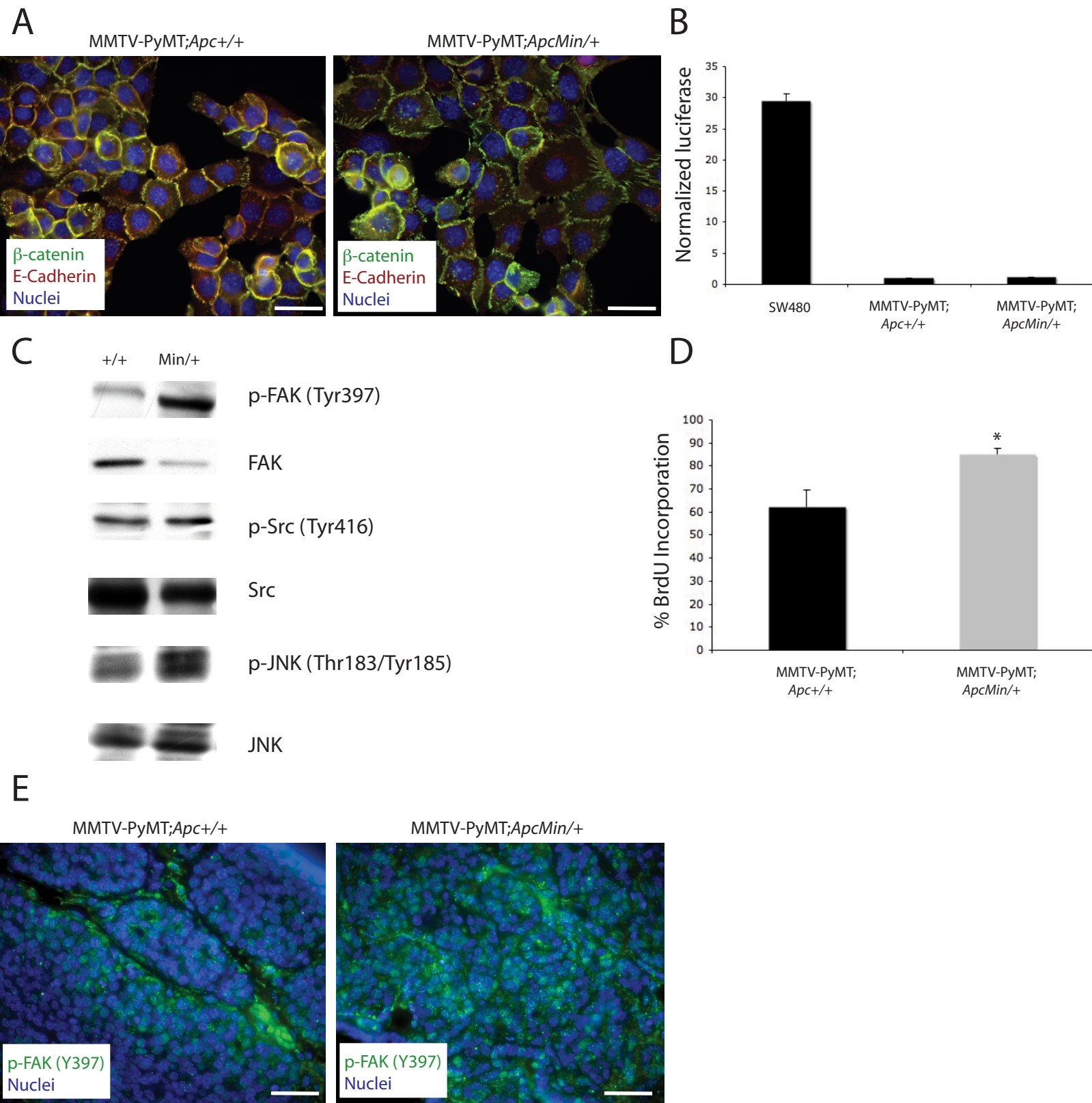

Supplement: Figure S3 — A second set of cell lines isolated from MMTV-PyMT; Apc+/+ and MMTV-PyMT; ApcMin/+ mice show similar phenotypes. (A) IF with a β-catenin antibody (green) and E-cadherin (red) antibody shows restricted localization of both proteins to cell-cell contacts in the control cells (left panel). In the Apc-mutant cells (right panel), junctional β-catenin and E-cadherin are observed but β-catenin is also localized in a punctate pattern in the cytosol or membrane. No nuclear accumulation of β-catenin is observed. Nuclei are stained with Hoechst (blue). Scale bars = 20 µm. (B) β-catenin/TCF reporter assays showed little basal activity in both cell lines. SW480 cells were used as a positive control. The data are shown as a ratio of normalized TOPflash∶FOPflash values. (C) Western blotting was performed on total cell lysates from the tumor cell lines with antibodies to detect total and phosphorylated forms of JNK, FAK and Src. Increased phosphorylation of JNK, FAK, and Src is observed in the MMTV-PyMT;ApcMin/+ tumor cells compared to control cells. (D) Tumor cell proliferation was assessed using BrdU incorporation and staining with an anti-BrdU antibody. Quantification shows that, consistent with the in vivo studies, MMTV-PyMT;ApcMin/+ tumor cells have enhanced proliferation compared to control cells. (E) IF with a phospho-FAK (Tyr 397) antibody (green) shows increased expression in the tumors from MMTV-PyMT;ApcMin/+ mice (right panel) compared to the control tumors (left panel). Nuclei are stained with Hoechst (blue). Scale bars = 20 µm. (PDF) [file pone.0029339.s003.pdf]
